# 3D phonon microscopy with sub-micron axial-resolution

**DOI:** 10.1038/s41598-021-82639-w

**Published:** 2021-02-08

**Authors:** Richard J. Smith, Fernando Pérez-Cota, Leonel Marques, Matt Clark

**Affiliations:** Optics and Photonics, Faculty of Engineering, University of Nottingham, University Park, Nottingham, UK

**Keywords:** Single-cell imaging, Imaging and sensing, Acoustics, Biophotonics, Photoacoustics

## Abstract

Brillouin light scattering (BLS) is an emerging method for cell imaging and characterisation. It allows elasticity-related contrast, optical resolution and label-free operation. Phonon microscopy detects BLS from laser generated coherent phonon fields to offer an attractive route for imaging since, at GHz frequencies, the phonon wavelength is sub-optical. Using phonon fields to image single cells is challenging as the signal to noise ratio and acquisition time are often poor. However, recent advances in the instrumentation have enabled imaging of fixed and living cells. This work presents the first experimental characterisation of phonon-based axial resolution provided by the response to a sharp edge. The obtained axial resolution is up to 10 times higher than that of the optical system used to take the measurements. Validation of the results are obtained with various polymer objects, which are in good agreement with those obtained using atomic force microscopy. Edge localisation, and hence profilometry, of a phantom boundary is measured with accuracy and precision of approximately 60 nm and 100 nm respectively. Finally, 3D imaging of fixed cells in culture medium is demonstrated.

## Introduction

Elasticity is being increasingly regarded as an important component of tissue and cell biology^1–5^. Conventional methods to measure elasticity observe deformation induced by a known force which is typically applied mechanically. This present challenges for biological cells in the form of invasiveness and low resolution. Ultrasonic methods such as photoacoustics (PA) or scanning acoustic microscopy (SAM) offer a non-invasive alternative. However, these techniques also exhibit limitations: for instance, the maximum resolution achievable by SAM on living cells is below that needed to resolve sub-cellular features^6^. Photoacoustics combines optics and ultrasound to map the optical absorption of cells and tissue with optical resolution^7^. However, at the micro scale, not all sub-cellular components provide sufficient optical absorption to create a photo-acoustic signal.

Brillouin light scattering (BLS) is an emerging method for cell imaging^8–12^ which offers elasticity-related contrast, optical resolution, compatibility with conventional light microscopy and label-free operation. Several forms of BLS scattering have been reported recently including spontaneous^13^ or stimulated^14^. However, these methods exhibit optical resolution, interference from substrates and often require calibration with a reference signal.

Picosecond laser ultrasound^15,16^ (PLU) is a technique traditionally used to observe ultra-fast acoustic phenomena. PLU can also access BLS through interferometry by resolving, in time, the propagation of a laser generated coherent phonon field^17^. This is commonly referred as time-resolved Brillouin scattering (TRBS). The TRBS signals, typically at GHz frequencies, have a wavelength shorter than that of visible light. This offers an opportunity for imaging with elasticity-related contrast and potentially super-optical resolution.

These features have excited interest in using TRBS for measuring^18–20^ and imaging^21–23^ biological cells. Recent works have demonstrated the estimation of elastic properties^20^ and 3D profiles^23^. Nevertheless, working with live or fixed cells in media is challenging while dehydrating cells, is easier, causes loss of 3D information. Alternatively, other PLU methods have been used based on acoustic reflectometry^22,24^ or thermal properties^25^. However, these are limited to the vicinity of the opto-acoustic/thermal transducer. In previous works, we have reported advances in TRBS for cell imaging in the form of transducer design and experimental configuration which we call *phonon microscopy*^26,27^. These led to enhanced SNR which can benefit acquisition speed or biocompatibility^28^.

This paper reports the first experimental characterisation of phonon resolution in the axial direction and the ability to produce 3D images of biological cells in culture medium with sub-micron resolution. By validating with atomic force microscopy, we show that the location of an object boundary can be detected with accuracy greater than the resolution provided by the phonon wavelength.

## TRBS for in-depth imaging

In time-resolved Brillouin scattering (TRBS), the amplitude and axial position of a laser-generated coherent phonon field is determined via Brillouin scattering (see “Methods” section). Here the frequency of the time-resolved signal is the *Brillouin frequency*:

1 $$\begin{aligned} f_B=\frac{2\nu n}{\lambda _o} \cos(\theta ), \end{aligned}$$

where $\lambda _o$ is the optical probing wavelength, $\nu$ the speed of sound, *n* the refractive index and $\theta$ the incident angle. If the measured volume is not homogeneous, then $f_B$ is a function of time where the spatial location of such variations is given by the speed of sound $z=\nu /t$. Hence, resolving temporal variations of $f_B$ enables in-depth imaging^23,28^.

From a theoretical point of view, where there is no a priori information, at least one complete cycle is needed to measure the frequency of a sinusoidal signal (such as the TRBS signal). Processing methods to recover changes in frequency will therefore have a finite transition width to a step change in frequency. The edge response ultimately determines the resolution of the technique. In previous work^28^, we have used a numerical model to estimate that the achievable axial resolution is half the edge response and a function of the optical probe wavelength $\lambda _o$ and the refractive index *n*:

2 $$\begin{aligned} z(min)= N \lambda _o / 4 n, \end{aligned}$$

where the wavelet bandwidth (*N*), is an integer number that defines the number of complete cycles used to calculate the frequency of the TRBS signal. This expression however, lacks experimental validation.

**Figure 1:**
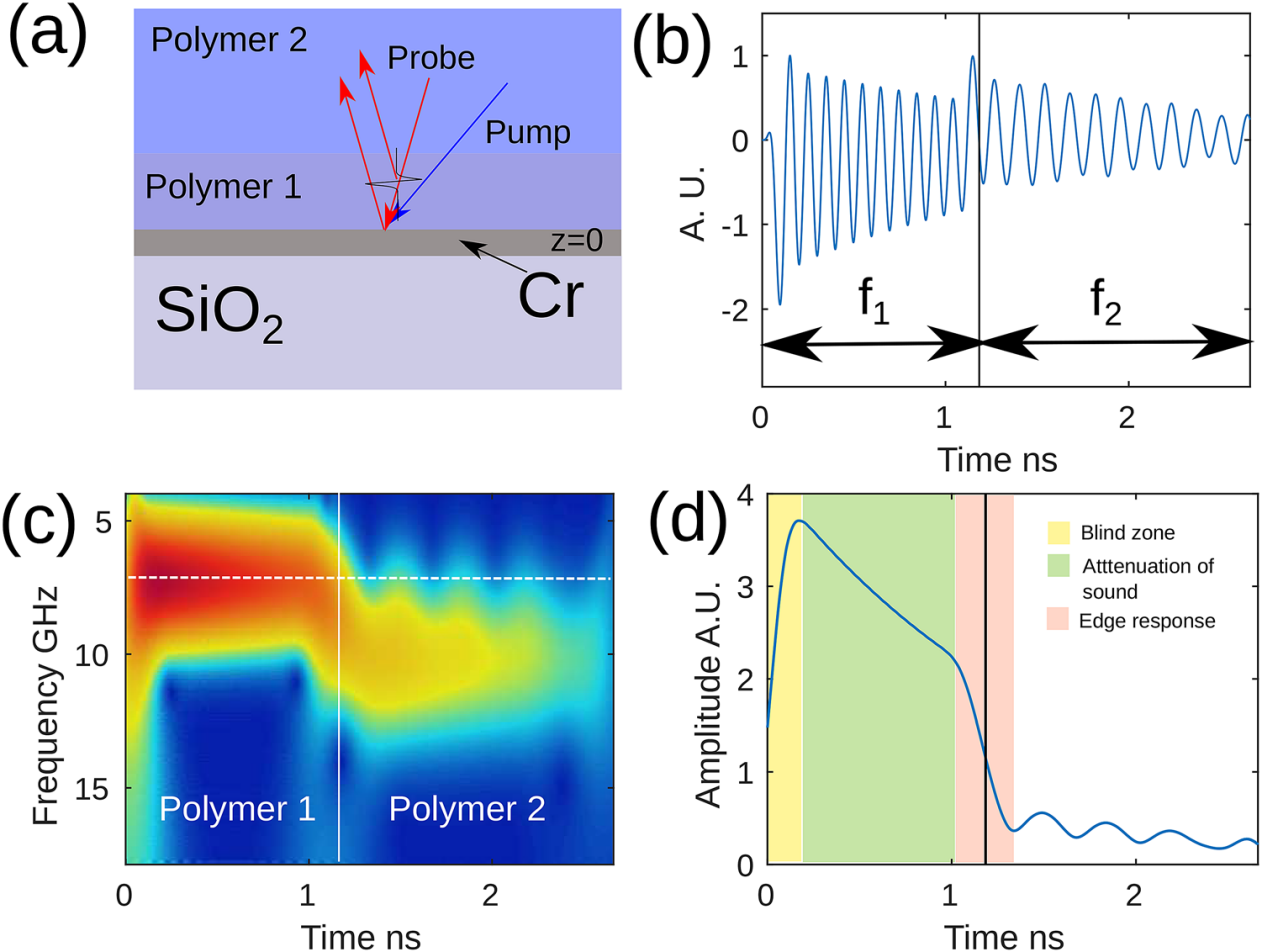
Simulated TRBS signal. (**a**) Model geometry for the simulated trace. 30 nm Cr film at 0 $\upmu$m position with two polymer layers on top. (**b**) Simulated modulation depth $\delta R/R$ from geometry in (**a**) showing a signal with two main frequency components $f_1$ and $f_2$ related to polymers 1 and 2 respectively. (**c**) Wavelet transform of signal simulated from geometry in (**a**). It shows the change in wavelet frequency vs time as sound propagates from polymer 1 to polymer 2. (**d**) Amplitude of wavelet transform at the frequency for polymer 1 (horizontal dashed line in (**c**)) showing three zones including sound attenuation and edge response.

Figure 1 shows a simulated TRBS signal (see “Methods” section). The sample comprises of a fused silica substrate, a thin chrome film (30 nm thick, z = 0) where the pump laser-generated phonon field originates and then propagates through two polymer layers with velocities $\nu _1=2500$ m/s and $\nu _2 = 1900$ m/s (see Fig. 1a). The pump and probe beams are incident from the top and the reflected optical intensity is calculated. The signal arises from the interference of the sound scattered light with the directly propagating light as indicated by the red arrows. An example waveform is shown in Fig. 1b where the observed oscillation is effectively an interference pattern whose frequency is the Brillouin frequency $f_B$. The propagation of the phonon field through the two polymers, which have different sound velocity, lead to different Brillouin frequencies ($f_1,f_2$).

To obtain in-depth information, a wavelet transform of the TRBS signal was used^29^ (see “Methods” section). The wavelet allows the recovery of the instantaneous frequency along the time trace. This leads to the reconstruction of the object in-depth due to two reasons: firstly, the TRBS signal frequency (the Brillouin shift) is related to the material properties (sound velocity and refractive index) and hence its change indicates a change in the volume being imaged. Secondly, in TRBS the signal is generated by propagating sound and hence the frequency at a given time is originated at a spacial position z = $\nu *t$ where $\nu$ is the sound velocity on the medium obtained from Eq. (1). An illustration of this is shown in Fig. 1c where the resultant wavelet amplitude changes in frequency as the sound wave propagates from polymer 1 to polymer 2. Thus an object imaged using TRBS can be reconstructed in the three dimensions.

The amplitude of the wavelet for the frequency $f_1$ for polymer 1 is shown in Fig. 1d. There are three components of the wavelet signal: (1) a blind zone which occurs when trace is not completely overlapping the wavelet window and this zone is small, (2) the sound attenuation zone which is a relatively slow decay of amplitude related to material properties and (3) the edge response which is the rapid change in amplitude related to the transition between materials. These components are key to understand the experimental results presented in the next section.

## Experimental edge response

**Figure 2:**
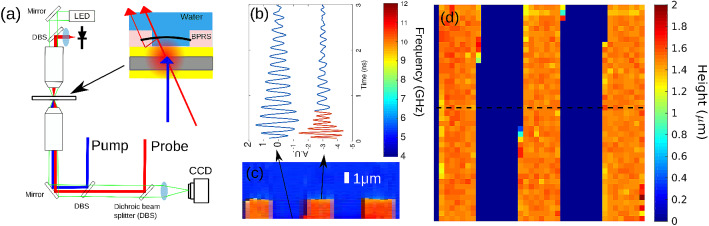
(**a**) Experimental schematic showing the pump and probe laser beams being delivered from the microscope and scattered light detected in transmission, inset shows the polymer sample and thin film transducer arrangement. (**b**) Example traces from water and polymer traces from (**c**) where the polymer trace clearly shows a change of frequency after $\sim$ 0.6 ns. (**c**) Cross section of the height map (see dashed line in (**d**)) for a BPRS grating. (**d**) The height map of the BPRS grating obtained by resolving the Brillouin frequency against time.

A phonon microscope was used to record the TRBS signals from a patterned polymer film (see “Methods” section) using water as the surrounding medium. Figure 2a shows the experimental arrangement based on an inverted microscope where the probe light is collected in transmission. The spin-coated film serves as a sharp edge which provides means to measure the edge response and hence the axial resolution.

Samples of the obtained TRBS signals are shown in Fig. 2b. Two frequency components exist in the signals: low frequency coming from the water (blue, n = 1.33) and high frequency coming from the BPRS (red, n = 1.6). By recovering the frequency against time using wavelet analysis (see “Methods” section), the temporal location of the transition can be found and with this, a spatial vector can be built. This vector is constructed using the sound velocity from each medium (obtained from Eq. (1)) and the temporal location of the transition. This ultimately leads to the reconstruction of the grating in 3D which reveals the thickness of the film of 1.48 ± 0.1 µm (see Fig. 2c,d). From this result, the precision of the localisation of the boundary, is estimated to be 100 nm. The resolution in this case, for N = 4, is $\sim$ 490 nm. The precision in the determination of the height is directly related to the variation on $f_B$ which is an attenuating signal. Hence a taller object would exhibit lower precision due to the loss of signal amplitude (and SNR) with *z*.

**Figure 3:**
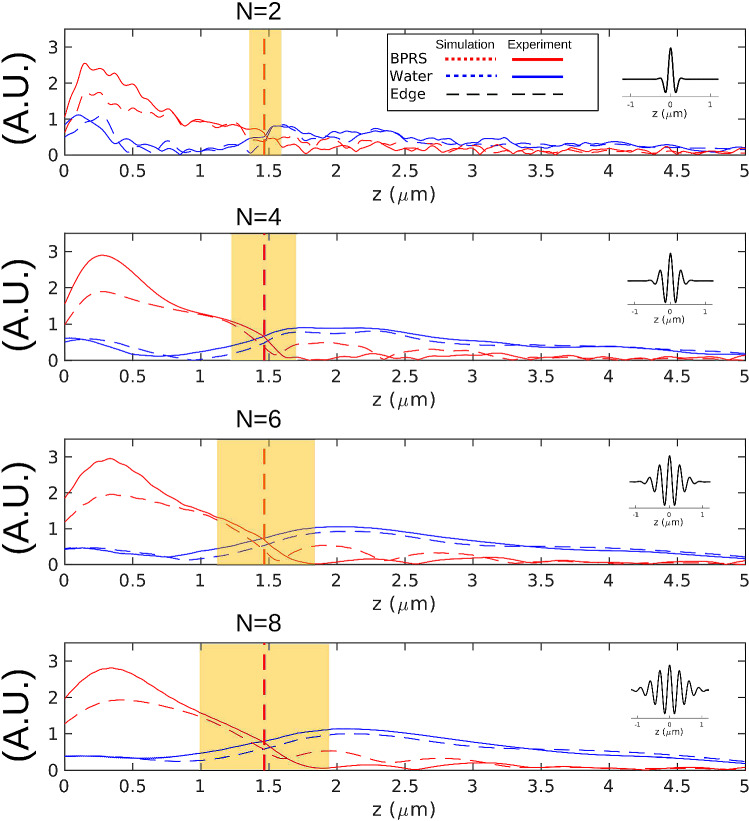
Wavelet analysis of simulated (dashed) and experimental (solid) polymer-water transition for different wavelet bandwidths. Insets show the resulting wavelet for N =2, 4, 6, and 8 which corresponds to (**a**–**d**). Edge response increases with greater bandwidth but precision remains similar as signal to noise increases. The transition point is accurately identified for each case at the point that the amplitudes of both frequency components (water and polymer) are the same.

Figure 3 shows the wavelet analysis for an edge response extracted from the dataset presented in Fig. 2. The amplitude of the wavelet as a function of *z* for the two detected frequencies (water and BPRS) is plotted for simulation and experiment and the match is good. The boundary between the two materials is found (vertical dashed line) by locating when the amplitude of the wavelet response at the either the water or polymer frequency is half way through the edge response. This is achieved by finding the end of the response by a threshold and then calculating its centre from the known material and wavelet parameters. The experimental edge response matches the theoretical wavelet bandwidth of 2$z_{min}$ (see yellow fringes). It must be noted that the amplitude of the wavelet reflects both sound attenuation and edge response as shown in Fig. 1d.

A trade-off between resolution (edge response width) against noise is observed as the wavelet window changes in size. The shorter the wavelet function, the narrower the edge response is, however noise increases. Despite the reduction of resolution with increased wavelet bandwidth, the precision of the localisation of the edge remains similar. However, in the case of multiple edges, it would not be possible to resolve them if their separation is below the resolution ($z_{min}$). To determine the accuracy of the localisation of the edge though, it is necessary to compare the TRBS measurements with an alternative method as shown in the following section.

## Cell phantoms

Two different cell phantom samples were made by melting polystyrene (PS) or a mixture of PS and Poly-methyl methacrylate (PMMA) microspheres onto a transducer substrate (see “Methods” section). The phantoms were mounted in a chamber so that a liquid surrounding medium could be used during the experiments. TRBS signals were recorded at each scan location and processed using wavelet transforms to extract 3D information.

**Figure 4:**
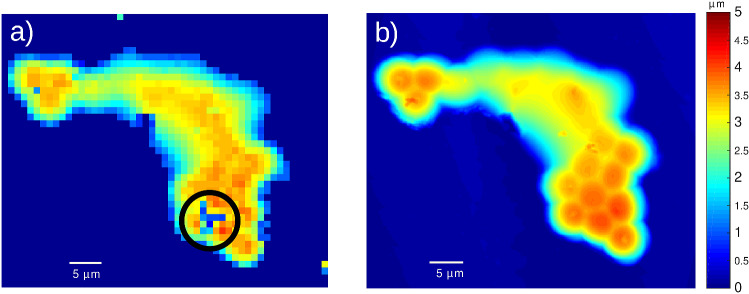
3D imaging of single material cell phantom. (**a**) Profile of the polystyrene phantom sample obtained using TRBS where ultrasound detects a sub-surface defect (circle) (**b**) profile obtained by AFM, which shows good agreement with the acoustic method ($\sim$ 60 nm).

The obtained profile of the first cell phantom (PS only), presented in Fig. 4a, shows that some of the polystyrene beads have melted more than others and that the heights vary from a few microns at the edges to around 4.5 microns at the thickest part. The beads were 5 microns in diameter before they were melted so this seems reasonable and is validated with an AFM profile image of the same phantom which agrees remarkably well (see Fig. 4b). The acoustics are affected by subsurface defects (see square in Fig. 4a) that appear as a no transition pixel. Accuracy was estimated by calculating the mean absolute difference between the two height maps presented in Fig. 4. From this operation, the accuracy was estimated as $\sim$ 60 nm. Accuracy in this case is significantly smaller than the TRBS resolution obtained using Eq. (2) (740 nm @ N = 6, n = 1.58).

**Figure 5:**
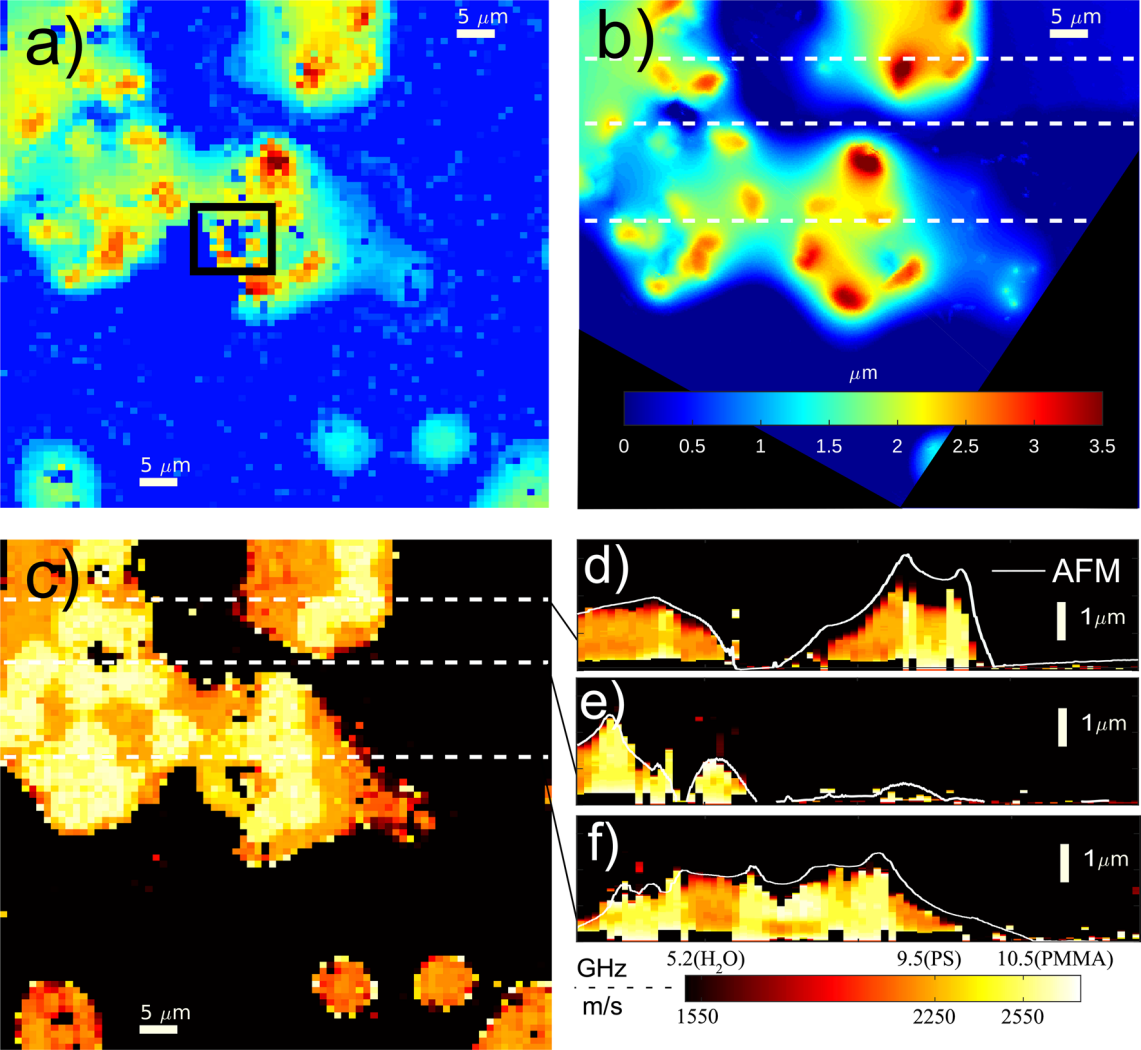
3D imaging of a two-polymer cell phantom. (**a**) thickness map of obtained using TRBS. The square region shows the presence of a subsurface defect (**b**) Profile of the same sample obtained using AFM. (**c**) Velocity map obtained at *z* position of 0.8 microns and applying refractive index information for each material, (see Eq. (1)) where the water, PMMA and PS can be distinguished by the black, yellow and orange colours. (**d**–**f**) Cross-sections obtained using wavelet analysis from the dashed lines in (**c**) showing the variation in sound velocity given by the two the materials in the phantom. The cross-sections are overlaid with the AFM height information showing a good match.

Imaging of a second phantom sample made up of two materials (PS and PMMA) is shown in Fig. 5. The agreement between the height maps, obtained acoustically (see Fig. 5a) and with AFM (see Fig. 5b), is very good. Similar to the previous phantom, a sub-surface defect prevented propagation of the phonon field (see square region of Fig. 5a).

The presence of two materials within the phantom shown in Fig. 5 provides variation in material properties below the surface. In this scenario, wavelet analysis resolves these variations in the form of changes in frequency ($f_B$) against time. However, as the refractive index of the two materials is known, the resulting 3D object is expressed as variations of the sound velocity against space (see “Methods” section) to produce an image purely based on elastic properties. Figure 5c shows the 2D velocity map of the phantom at as a single *z* position where three distinct sound velocities are seen in black (water), yellow (PMMA) and red (PS). The PMMA areas correlate with the thickest parts of the sample which imply these beads did not melt down as much as the PS ones. Three cross-sections obtained from wavelet analysis (from the dotted lines in (c)) are shown in Fig. 5d–f with their AFM profiles overlaid—phonon imaging clearly distinguish between different materials along the measured volume at high resolution. This is a clear advantage over AFM since besides profiling, it can also map an object beyond its surface and discern materials based on their sound velocity.

## Cell imaging

**Figure 6:**
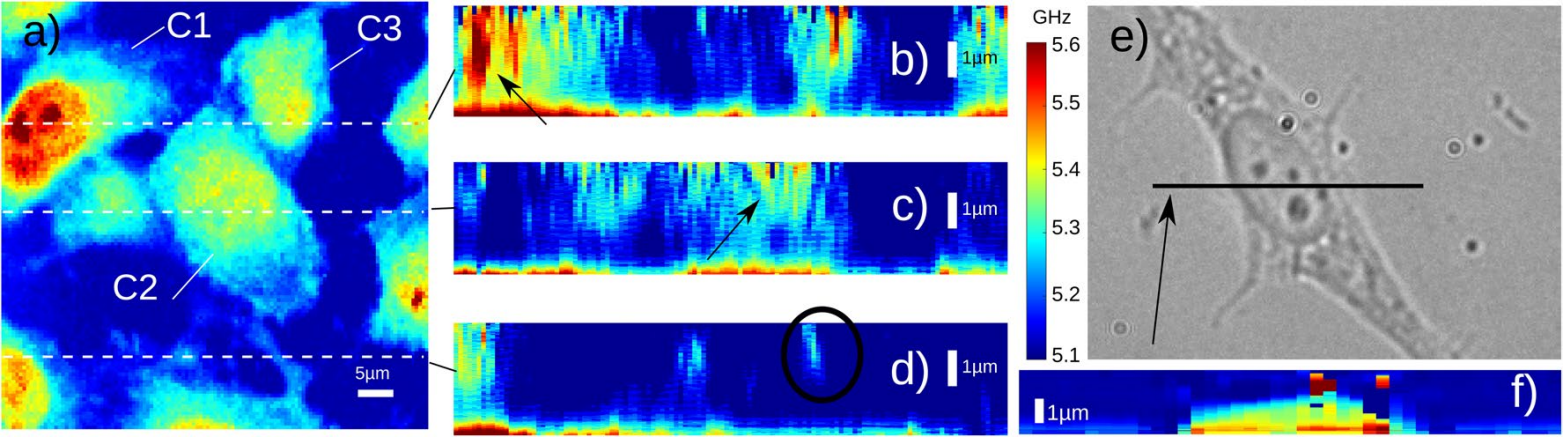
3D imaging of a fixed 3T3 cells. (**a**) Acoustic image showing the dominant frequency within the measurement depth, here a number of cells are visible within the scanned region with the nucleus showing higher frequency response for cell C1. (**b**–**d**) Cross sections from the dotted lines in (**a**) expanded 5 times with respect to scale bar in (**a**) (5 $\upmu$m). Different cells show the nucleus at different height. Filopodia is not attached to the substrate and suspended at a approximately 2 $\upmu$m. (**e**–**f**) Line scan with 10 times additional signal averaging that provides enough SNR to see through a whole 3T3 fibroblast cell. All images used the same colour bar.

An opto-acoustic transducer was coated on sapphire coverslips^27^ and 3T3 fibroblast cells were seeded and fixed (see “Methods” section). TRBS signals were recorded but this time the wavelet analysis was limited to the Brillouin frequency only. This is due to the lack of refractive index information at the adequate scale. Instead, the temporal vector was approximated to a spatial vector by considering a constant sound velocity (1550 m/s). The 2D frequency map, obtained averaging the z space, of a 60 $\upmu$m$^2$ scanning region is shown in Fig. 6a. There are three complete 3T3 cells in the field of view (marked as C1, C2 and C3) for which C1 has clearly higher frequency for the nucleus and shows some internal features. However, this level of contrast for the nucleus is not visible for cells C2 and C3.

Figure 6b–d shows cross-sections obtained with wavelet analysis from the TRBS data on Fig. 6a where the vertical scale is expanded 5 times with respect to that of Fig. 6a. Compared to phantoms, sound in the cell attenuates faster and hence the SNR drops before imaging the whole object. The cross-section through C1 shows the nucleus close to the substrate (in the z direction) and hence gives a strong response. Figure 6c shows a slice through C2, here the nucleus is axially away from the substrate so is not imaged as it is just outside the imaging depth of the experiment. This is not obvious from the 2D map as the axial location of the features has a strong influence on the average Brillouin frequency because the signal strength decreases with depth. Being able to remove this ambiguity is important for repeatability and confidence in these measurements.

Figure 6d shows the axial location of filopodia (cell adhesion projections, see circle) at approximately 2 $\upmu$m showcasing the potential of the technique for resolution and contrast. Finally, Fig. 6e shows the location of a single line scan in Fig. 6f (averaging $\times$ 10 longer) where the TRBS signal is detected through the whole cell—this is important as it demonstrates that sound propagation at the GHz frequencies has enough penetration depth to image single cells in culture, demonstrating complete through cell imaging.

## Discussion

We have presented recent advances of time-resolved Brillouin scattering (TRBS) for 3D imaging of biological cells in aqueous media. We demonstrate and validate imaging of the Brillouin shift with 490–740 nm axial resolution and single edge localisation with high accuracy ($\sim$ 60 nm) and precision ($\sim$ 100 nm). This was achieved with relatively low NA (0.42–0.6) and without confocal arrangements.

The axial resolution of the optical instrument used to take the data on the cell phantoms is $\sim$ 3.5–7 $\upmu$m. The axial resolution of acoustic data obtained with this instrument depends on the acoustic wavelength. As discussed previously in “TRBS for in-depth imaging” section, the axial resolution depends on the phonon wavelength which is used for the measurement. The probing optical wavelength determines which phonon wavelength scatters the light by satisfying the Bragg wavelength condition ($\lambda _{a} = \lambda _{o} / 2n$). The optical wavelength in the media ($\lambda _{o}/n$) therefore sets the minimum size of the axial resolution (one cycle). As most time-frequency methods require more than one cycle (N > 2) to determine the frequency, so the axial resolution scales with the number of cycles used as shown in Eq. (2). For the case of wavelets, the bandwidth parameter determines the number of cycles and therefore the axial resolution^28^. In this paper, the wavelet parameters used a bandwidth setting of N = 4–6 to determine the frequency, giving a resolution of $N\lambda _o /4 n \sim$ 490–740 nm which is approximately 5 to 10 times smaller than the optical axial resolution of the system used for the measurements.

One advantage of using the acoustics to access the depth information is that a full time resolved measurement is obtained at each point, and so there is no need to successively scan the sample with different focal positions as is the case for optical sectioning methods (such as confocal imaging). This is very advantageous; the sample is only exposed to light once for each measurement point, the depth information is obtained at the same time and so the measurement is robust to changes in the sample during the measurement. Additionally, the optimal sectioning strategy can be chosen post scan depending on the achieved SNR and the velocity contrast of the sample.

During the extraction of boundary locations, there is an in built assumption that the impedance mismatch of the materials is small, which is the case for cells and the polymers used in this study, this means that each temporal position corresponds to a single spatial position. Where the mismatch is high and gives rise to large reflections this will make the transitions more complex and this simple processing will fail.

One trade-off with this processing technique is that to improve the axial resolution, the frequency resolution is made poorer as fewer cycles are used in the calculation of the frequency. Loss of frequency resolution translates into an increase in error on the recovered acoustic velocity. The standard deviation of the sound velocity within the water measured from the sample shown in Fig. 4 was 6 m/s considering the whole axial volume which is only approximately a 0.4% variation. The error on the estimation of the signal frequency (or sound velocity) generally increases with depth due to the signal attenuation. So, for deep imaging, more averages need to be taken to maintain sufficient SNR to see the difference between objects. For instance, in the same dataset, the standard deviation of the velocity of the sections before 1 ns is approximately 37 m/s ($\sim$ 2.4%) while after 3 ns is 50 m/s ($\sim$ 3.3%).

The lateral resolution is limited by the optical pump spot size. This is currently around 0.5 and 1 micron for the phantoms and cells respectively. Using higher NA objective lenses could improve this further but this is still limited by diffraction. To move beyond the optical diffraction limit would require, for instance, moving away from planar transducers to implement spherical nano-particles^30,31^.

Being able to obtain the Brillouin frequency in three dimensions enables 3D characterisation of cells with contrast related to their mechanical properties. However to perform quantitative measurements of standard elastic properties, it is necessary to know two other parameters such as the local refractive index and mass density. To obtain either of these in 3D at the scales available to the acoustics is challenging but it has been shown to be possible^32^. The all optical nature of the phonon microscope means that other optical techniques such as phase tomography or ptychography can be incorporated into the instrument to achieve quantitative characterisation of the sound velocity.

We have demonstrated that ultrasonics combined with Brillouin scattering offers an attractive route to high resolution 3D imaging. The ability to time resolve the signals enables reconstruction of objects, including biological cells, in three dimensions with high axial resolution and without confocal arrangements. High frequency ultrasound has a number of attractive features, it is label free, non invasive and compatible with imaging living cells. All these capabilities together promise great potential for enabling novel research in life sciences and health care.

## Methods

### Experimental setup

Picosecond laser ultrasound^15,16^ (PLU) uses two short ($\sim$ 150 fs, 100 MHz repetition rate) laser pulses to generate and detect high frequency acoustic waves. The pump pulse (390 nm) is absorbed by the sample itself or by a transducer layer (often a metal film); the absorbed light causes rapid heating and via thermal expansion launches an acoustic wave packet into the sample. The technique is widely used to look at thin films^33,34^ or micro/nano structure dynamics^35–38^. In this work an objective lens with NA of 0.42 was used for imaging of phantoms and 0.6 for biological cells.

The TRBS signal^17^ arises due to the interference of the reflected or transmitted probe (780 nm) laser beam with a portion scattered from the acoustic wave packet propagating in the sample (see Fig. 1a). As the wave moves, the phase of the scattered component changes relative to the non-scattered light producing an oscillating signal.

Figure 2a shows a typical experimental configuration for time resolved Brillouin scattering measurements for transparent media. The pump and probe laser beams are delivered from an inverted microscope onto the transducer substrate. The transducer is specially designed for phonon imaging and consist of a gold and indium tin oxide structure^26,27^. The pump probe method used is synchronised via an ASOPS configuration^39^ which have greatly increased the data acquisition speed allowing extremely weak signals to be detected in a reasonable time or allowing sufficient points to be captured so that images can be recorded.

### Modelling

With the purpose of characterising axial resolution and assess signal processing, we have modelled this process to produce exemplar waveforms as previously reported^28^. The model is based on^16^ and calculates the thermo-elastic generation process for a laser pulse being absorbed by the sample, which allows interferometric or reflectivity signals to be obtained.

The optical absorption is calculated using Fresnel coefficients, giving the absorption of the laser pulse in each layer of the sample. This is then converted to heat through the thermal properties of each layer. The thermal expansion of heated regions then leads to an initial stress in the material which is then propagated through the sample. The detection process uses a Green’s function approach to calculate how the probe laser beam is reflected from the sample structure, the displacement and the changes in reflectivity due to the propagating strain is calculated.

### Signal processing

Wavelet transforms were calculated for each time trace (or each pixel on the image) at each time position, the equivalent centre frequency for the wavelet number with the highest amplitude was stored. This results in a x, y, t matrix of Brillouin frequencies. From this matrix, several conversions are possible: if the refractive index is known, it can be converted to sound velocity by: $f_B = 2 n \nu / \lambda _{o}$ and subsequently the temporal axis can be converted to spatial by $z=\nu *t$. For the case of the cells presented in Fig. 6, the matrix was left as a frequency matrix, however the temporal axis was converted to spatial by approximating the sound velocity of the whole matrix to that of water.

The wavelet method was used because it has the advantage that the number of cycles used for each wavelet number is fixed and so is the frequency resolution. This means that the time resolution scales with frequency automatically as it depends on the number of cycles used. The wavelet transform is defined as:

3 $$\begin{aligned} W_{\psi }(a,b)= \frac{1}{\sqrt{a}} \int _{-\infty }^{\infty } f(t)\psi \Bigg (\frac{t-b}{a}\Bigg )dt, \end{aligned}$$

where $\psi$ is the mother wavelet, *a* denotes the wavelet dilation and *b* is the time shift of the wavelet. The mother wavelet used here is the Morlet which is a complex exponential multiplied by a Gaussian window:

4 $$\begin{aligned} \psi (\eta )= \pi ^{-1/4}e^{i\omega _0\eta }e^{-\eta ^2 /2}, \end{aligned}$$

where $\psi$ is the value of the wavelet at non-dimensional time $\eta$ and $\omega _0$ is the wavenumber. Matlab’s wavelet analysis toolbox and a complex Morlet transform (*cmor*) were used to extract $f_B(t)$ from the time-resolved signal. The width of the transform is defined by the bandwidth parameter (*a*).

### Fabrication

#### Polymer pattern

A BPRS photoresist pattern was fabricated using standard photolitography techniques. A transducer coated coverslip was spin coated with BPRS-100@4000 rmps. Then cured at 90 $^\circ$C for 5 min on a hot plate. The spin coated film was then exposed through a mask for 8 s using a Karl Suss MJB3 mask-aligner (7 mW/cm$^2$) and developed for 25s using AZ400K developer 8:1 diluted with distilled water.

#### Polymer phantom samples

A glass coverslip etched with a reference grid was cleaned with acetone and isopropanol and dried with N$_2$. It was then coated with a three layer thin film stack to act as a generation transducer^27^. The stack consisted of 20 nm gold, 140 nm ITO and 20 nm of gold (for all the experimental results presented in this work). Polystyrene (PS) microspheres 10% wt (Bangs Laboratories Inc., USA) with 5 micron diameters were drop coated onto the substrate which was spun-cast (spin-coater model ws-400 bz-6npp, Laurell Technologies Corporation, USA) for 30s at 750 rpm and placed into an oven @ 245 $^{\circ }$C for 30–45 min to partially melt the microspheres.

For the second sample, polystyrene (PS) and poly(methyl methacrylate) (PMMA) microspheres were prepared over an etched gridded glass coverslip (Electron microscopy sciences #72265-50). The glass slide was initially cleaned with acetone and isopropanol and dried with N$_2$. A transducer device was fabricated over the coverslip surface. A diluted solution (1:500; v:v) of 10 $\upmu$m PMMA microspheres 10$\%$ wt (Bangs Laboratories Inc., USA) in water and drop coated over the Au surface. The sample was spun-cast for 30 s at 750 rpm (spin-coater model ws-400 bz-6npp, Laurell Technologies Corporation, USA) and placed in an oven @245 $^{\circ }$C for 5 min. The sample was removed from the oven and a diluted solution (1:500; v:v) of 5 $\upmu$m PS microspheres 10$\%$ wt (Bangs Laboratories Inc., USA) in water was drop-coated over the surface. The sample was spun-cast for 30 s at 750 rpm and placed back in an oven @ 245 $^{\circ }$C for 30–45 min. These two polymers have different refractive indices and acoustic velocities and so will have different Brillouin frequencies.

#### Cell preparation

Sterile transducer coated sapphire coverslips (25mm diameter) were coated with Poly-L-lysine solution (0.01% (w/v): Sigma-Aldrich; P4707) to promote cell adhesion. Coverslips were each seeded with 2.5 $\times$ 10$^5$ mouse embryonic fibroblast NIH–3T3 cells (ATCC$\circledR$ CRL-1658, USA). These were cultured in DMEM (Sigma-Aldrich; D6421) with 10% (v/v) Fetal Bovine Serum (FBS: Sigma-Aldrich; F7524), 1% (v/v) penicillin-streptomycin (Sigma-Aldrich; P0781) and 1% (v/v) L-glutamine (200mM: Sigma-Aldrich; G7513) at 37 $^\circ$C/5% CO2 for 24h.

NIH-3T3 cells were fixed to the gold coated coverslips using 4% (v/v) formaldehyde solution (Sigma-Aldrich; 1004968350) for 30 minutes and washing 3$\times$ with Dulbecco’s Phosphate Buffered Saline (PBS: Sigma-Aldrich; D8537). Coverslips were stored at 4 $^\circ$C in PBS until required.
